# 

**Published:** 1873-04

**Authors:** 


### CONTENTS:

### ORIGINAL COMMUNICATIONS.

Article I.—Valedictory Address. By W. W. H. Thackston, M. D., D. D. S. .......... 529

Article II.—Is Woman Adapted to the Dental Profession. By Miss Emilie Foeking, D.D.S. 542

Article III.—Sound Practice, Sound Philosophy. (Continued). By M. S. Dean, D.D.S. 553

### SELECTED ARTICLES.

Article IV.—Diseases of the Gums. By Dr. H. S. Miller .......... 558

Article V.—Death from Chloroform .......... 560

Article VI.—The St. Louis Dental Society .......... 563

### MONTHLY SUMMARY.

Death from Ether .......... 566

The Cure of Stammering .......... 566

Wear and Repair of the Brain .......... 567

Neuralgia .......... 568

Ether against Chloroform .......... 568

Pill for Neuralgia .......... 568

Five Temporary Teeth at Forty .......... 569

Bleached Tincture of Iodine .......... 569

### BIBLIOGRAPHICAL.

Dental Caries and its Causes. By Drs. Leber and Rottenstein.
Tanslated by Thomas H. Chandler, D. D. S. 570

### EDITORIAL DEPARTMENT.

Death Following the Inhalation of Nitrous Oxide Gas .......... 571

Wedl's Pathology of the Teeth .......... 573

Woman's True Rights .......... 574

The Medical Diploma Traffic .......... 575

The Rubber Question .......... 576

Osborn's Tongue Holder and Duct Compressor .......... 576

Women as Doctors .......... 576

